# Macronutrient, immunoglobulin a and total antioxidant capacity profiles of human milk from 1 to 24 months: a cross-sectional study in Thailand

**DOI:** 10.1186/s13006-020-00333-5

**Published:** 2020-10-30

**Authors:** Krongporn Ongprasert, Jetsada Ruangsuriya, Rungnapa Malasao, Ratana Sapbamrer, Pikul Suppansan, Pisittawoot Ayood, Kulnipa Kittisakmontri, Penprapa Siviroj

**Affiliations:** 1 Department of Community Medicine, Faculty of Medicine, Chiang Mai University, Chiang Mai, Thailand; 2 Department of Biochemistry, Faculty of Medicine, Chiang Mai University, Chiang Mai, Thailand; 3 Maharaj Nakorn Chiang Mai Hospital, Faculty of Medicine, Chiang Mai University, Chiang Mai, Thailand; 4 Department of Pediatrics, Faculty of Medicine, Chiang Mai University, Chiang Mai, Thailand

**Keywords:** Breastfeeding, Human milk, Immunoglobulin, Macronutrient composition, Prolonged lactation, Total antioxidant capacity

## Abstract

**Background:**

An extended duration of breastfeeding of up to two years is encouraged by many health authorities, but information regarding the composition of milk after one year postpartum is limited. The goal of this study was to determine the association between the duration of lactation and macronutrient contents, immunoglobulin A (IgA) levels and total antioxidant capacity (TAC) in human milk (HM), from 1 to 24 months postpartum.

**Methods:**

Cross-sectional milk samples were collected between January and April 2019 from mothers with healthy full-term children who had been lactating for 1 to 24 months. The HM was biochemically analyzed for protein and carbohydrate contents by colorimetric assays. The fat content was determined by capillary centrifugation, and the energy content was calculated from the results of centrifugation assays. IgA levels and TAC were determined by ELISA and a Trolox equivalent antioxidant capacity (TEAC) assay, respectively. Pearson’s correlation coefficient and Spearman’s rank correlation coefficient were used to determine associations between months of lactation and milk composition, and multiple regression analysis was used to assess associations between months of lactation and milk composition adjusted for relevant covariates. Differences were considered significant at *p* < 0.05.

**Results:**

One hundred eighty-four milk samples were analyzed. The month of lactation was positively associated with the fat concentration (B = 0.31, SE = 0.09, *p =* 0.001), energy content (B = 3.11, SE = 0.92, p *=* 0.001), and IgA (B = 4.17, SE = 1.08, *p <* 0.001) but negatively associated with the carbohydrate concentration (B = − 0.22, SE = 0.01, *p =* 0.04). No association was observed between the month of lactation and the protein concentration or TAC after adjustment for maternal age, maternal BMI, birth order, and breastfeeding frequency.

**Conclusion:**

The duration of lactation was found to be positively associated with the fat, energy, and IgA content in HM for up to two years postpartum, and negatively associated with carbohydrate concentration. More prospective cohort studies are needed to obtain evidence-based knowledge regarding the changes in HM composition throughout the course of lactation.

## Background

Human milk (HM) is widely accepted as an optimal food and provides essential components for the growth and development of infants. Apart from macronutrients and micronutrients, HM contains various non-nutritive bioactive compounds, including prebiotics, growth factors, hormones, and antioxidants, as well as components that protect against infection such as lysozyme, lactoferrin, oligosaccharide, and immunoglobulin A (IgA) [1–3]. In addition to supporting normal growth and development, breastfeeding offers numerous advantages, including psychological, economic, and environmental benefits. Recent advances in molecular biology techniques have shown that HM plays an essential role as an epigenetic modulator of gene expression in milk recipients and may positively impact life-long metabolic programming [4, 5].

A longer duration of breastfeeding is encouraged by the World Health Organization [6], which recommends exclusive breastfeeding for the first six months, along with continued breastfeeding for at least two years. The American Academy of Pediatrics has reaffirmed the recommendation of exclusive breastfeeding for approximately the first six months followed by continued breastfeeding as complementary foods are introduced with the continuation of breastfeeding for at least one year of life [7].

HM has been well established to be a dynamic fluid with a composition that continually changes throughout the lactation period. During the colostrum and transitional stage of lactation (within the first 10–14 days postpartum), the composition of breast milk undergoes remarkable changes. Mature milk gradually replaces transitional milk after approximately two weeks postpartum and remains relatively similar in its composition, with subtle changes occurring during the weaning period [8]. Although the composition of milk produced during the first six months postpartum has been widely reported, information on milk composition during the second year postpartum is limited and inconclusive due to small sample sizes, non-standardized sample collection protocols, and limitations associated with study designs. Moreover, IgA, which is the predominant immunoglobulin in HM, and the antioxidant capacity, which supports the immature immune system by neutralizing pathogens and removing free radicals, are rarely reported [9–11]. The goal of this study was to determine the association between the month of lactation and macronutrient composition, IgA content and total antioxidant capacity (TAC) of HM, from 1 to 24 months.

## Methods

### Study design

This cross-sectional study included 184 breastfeeding mothers who had been lactating for 1 to 24 months. Participants were recruited from January 2019 to April 2019 through study posters posted in the well-baby clinic and the lactation rooms of four hospitals in Chiang Mai City, Thailand. Participants were also recruited from a Facebook parenting group. After interested mothers contacted the study staff via telephone, they were asked a set of questions corresponding to the inclusion and exclusion criteria. Lactating mothers who had given birth to a full-term infant were recruited for this study. The exclusion criteria were as follows: (a) any underlying disease in the mother or her offspring, (b) maternal age under 18 years or above 40 years, (c) illiteracy in the Thai language, and (d) an inability of the mother to travel to our lactation room on her own. All eligible participants were then asked to set an appointment for milk collection. Participants completed a self-report paper-based questionnaire in the Thai language to collect information regarding maternal age, education level, first antenatal care (ANC) visit, gestational age, birth order, parental status, and breastfeeding frequency. The weight and height of each participant were measured before milk samples were collected. Before providing information and breast milk samples, all participants signed informed consent forms. The participants received no payments.

### Sample collection

Participants were required to provide milk samples in the lactation room of Maharaj Nakorn Chiang Mai Hospital, Nakornping Hospital, Health Promotion Hospital Region One and Lampang Hospital. To minimize possible circadian influences [9] and to ensure uniformity of the samples, all breast milk samples were expressed between 8:00 AM and 12:00 PM using a Lactina Electric Selection pump (Medela, Switzerland). The pump was left on for approximately 15 min or until no further milk could be expressed for at least five minutes. For storage, the samples were aliquoted into 1.5-mL microcentrifuge tubes and frozen at − 80 °C until further analysis. Samples collected for antioxidant activity measurements were stored at 0 °C and analyzed within 72 h to preserve the antioxidant activity.

### Biochemical analyses of human milk

#### Carbohydrate content

The total carbohydrate content in HM was estimated using a 3,5-dinitrosalicylic acid (DNS) solution prepared by solubilizing 1 g of DNS (Sigma, 128,848) in a 2 M NaOH (VWR Chemicals, 28,244.295) solution containing 30 g Na-K tartrate (VWR Chemicals, 27,068.233), and then deionised (DI) H_2_O was added to reach a total volume of 100 mL; this solution was referred to as the working DNS solution. The milk samples were diluted 25× with DI H_2_O, and 500 μL of each diluted sample was mixed with 500 μL of working DNS solution. The mixture was then boiled for five minutes and cooled down in running tap water. Then, 4 mL of DI H_2_O was added to each reaction, and the absorbance was read at 540 nm with a Synergy H4 Hybrid Reader (BioTek, USA). The concentration of carbohydrates in the milk was calculated from a D-lactose (Sigma, 61,345) standard curve with a concentration range of 0–100 mg/mL.

#### Protein content

The total protein content in HM was determined by Lowry’s method using Folin-Ciocalteu solution (VWR Chemicals, 31,360.264). The milk samples were diluted 100× with DI H_2_O, and 500 μL of each diluted sample was mixed with 2.5 mL of an alkaline solution and 250 μL of the Folin-Ciocalteu solution. The mixture was incubated at room temperature (RT) for ten minutes, and the absorbance was read at 650 nm with a Synergy H4 Hybrid Reader (BioTek, USA). The concentration of protein in the milk was calculated from a bovine serum albumin (GE Healthcare, K41–001) standard curve with a concentration range of 0–100 mg/mL.

#### Creamatocrit, lipid content, and energy conversion

The percentage of cream (creamatocrit) in HM was examined by capillary centrifugation followed by calculation of the lipid content and energy yield. The milk samples were individually loaded into each capillary tube to 4/5 of the tube capacity, and the filled tube was capped with clay. Then, the tubes were microcentrifuged (Hettich Haematokrit, Germany) for 15 min. The thickness of the cream (A) and the total solution heights (B) were measured. The creamatocrit was calculated as 100 × (A÷B), lipid content (g/L) as (creamatocrit × 5.57) - 3.08, and energy (kcal/100) as (creamatocrit × 5.57) + 45.13.

#### Immunoglobulin a (IgA)

IgA levels in HM were determined using a commercial ELISA kit (Aviva System Biology, OKEH00516) according to the manufacturer’s protocol. Briefly, the HM samples were diluted 200,000× in water as well as assay diluent buffer. Then, 100 μL of the diluted samples and the IgA standard were loaded into each well of an ELISA plate. The samples were incubated at 37 °C for two hours, and then the solution in each well was replaced with 100 μL of biotinylated IgA detector antibody. The samples were incubated at 37 °C for an hour, and the solution in each well was discarded and washed. An avidin-HRP conjugate mixture was added at 100 μL into each well and incubated at 37 °C for another hour. The solution in the well was discarded, and the plate was washed. Then, 90 μL of TMB substrate was added to each well, and the plate was incubated in the dark at 37 °C for 15 min. Finally, 50 μL of the stop solution was added to each well, and the plate was read at an absorbance of 450 nm with a Synergy H4 Hybrid Reader (BioTek, USA). The concentration of IgA in the milk was calculated from an IgA standard curve with a concentration range of 0–4000 pg/mL.

#### Total antioxidant capacity

TAC of HM was determined as the Trolox equivalent antioxidant capacity (TEAC) using ABTS solution, which was prepared by mixing two equal volumes of 0.768 g% of ABTS (AppliChem, A1088,0005) and 0.132 g% of K_2_S_2_O_8_ (VWR Chemical, 26,915.291). The mixture was incubated at RT for 12 h, and the working ABTS solution was made by diluting the stock solution 50× in DI H_2_O. Twenty microliters of HM sample was mixed with 2 mL of the diluted ABTS solution. The reaction ran for six minutes, and the absorbance was read at 734 nm with a Genesys 20 instrument (Thermo Scientific, USA). TAC in each HM sample was calculated using a Trolox (Sigma, 238,813) standard curve with a concentration range of 0–5 mM, and was reported as the millimolar Trolox equivalence.

### Statistical analysis

This was a cross-sectional study. The data are presented as descriptive statistics, including the mean, standard deviation (SD), frequency (n), percentage (%), median, interquartile range, and range. Pearson’s correlation coefficient and Spearman’s rank correlation coefficient were used to determine associations between months of lactation and milk composition. Participants were also divided into four groups based on breastfeeding duration. Kruskal-Wallis and Mann-Whitney tests were used to test differences in macronutrient and energy contents in breast milk by group, whereas one-way ANOVA post hoc tests and independent-sample T tests were used to test differences in IgA levels and TAC in breast milk by group. Finally, multiple linear regression analysis was used to assess the association between months of lactation and milk composition with maternal age, maternal body mass index (BMI), birth order, and breastfeeding frequency as covariates. Differences were considered significant at *p* < 0.05.

## Results

The participants were divided into four groups based on breastfeeding duration: 1–6 months (*n* = 43), 6–12 months (*n* = 47), 12–18 months (*n* = 50), and 18–24 months (*n* = 44). No significant differences between the groups were found with respect to demographic or sample characteristics (Table 1).

**Table 1:** Sample characteristics

	Breastfeeding 1–6 months (*n* = 43)	Breastfeeding 6–12 months (*n* = 47)	Breastfeeding 12–18 months (*n* = 50)	Breastfeeding 18–24 months (*n* = 44)	p-value
Maternal age, years (mean ± SD; median, P25^th^–75th) ^1^	32.6 ± 3.6, 32, 29–35	30.9 ± 4.6, 31, 28–35	32.2 ± 4.8, 32, 33–36	32.0 ± 4.5, 32, 29–37	0.28
Maternal BMI, kg/m^2^ (mean ± SD; median, P25^th^–75th) ^2^	23.2 ± 4.5, 22.5, 19.7–24.8	22.6 ± 4.8, 21.3, 19–25.1	22.3 ± 4.1, 21.6, 20.2–23.8	22.3 ± 3.3, 21.7, 19.8–25.1	0.78
Educational level (n, %)^3^					
Primary school	9 (20.9)	7 (14.9)	16 (32.0)	12 (27.3)	0.13
Secondary school/certificate	0 (0)	3 (6.4)	4 (8.0)	4 (9.1)	
Graduate degree	34 (79.1)	37 (78.7)	30 (60.0)	28 (63.6)	
First ANC visit, months (mean ± SD; median, P25^th^–75th) ^2^	2.0 ± 1.3, 2, 1–2	2.3 ± 1.6, 2, 1–3	2.3 ± 1.3, 2, 1–3	2.1 ± 1.2, 2, 1–3	0.78
Gestational age, days (mean ± SD; median, P25^th^–75th) ^2^	270.6 ± 6.4, 272, 266–273	271.3 ± 8.1, 272, 266–278	271.5 ± 6.4, 273, 266–275	272.3 ± 8.3, 273, 266–280	0.84
Birth order ^3^ (n, %)					
First born	27 (62.8)	31 (66.0)	33 (66.0)	25 (56.8)	0.71
Second born	15 (34.9)	15 (31.9)	13 (26.0)	16 (36.4)	
Third-fifth born	1 (2.3)	1 (2.1)	4 (8.0)	3 (6.8)	
Parental status, couple (n, %) ^3^	42 (97.7)	47 (100)	48 (96.0)	44 (100)	0.46
Breastfeeding frequency, times per day (mean ± SD; median, P25^th^–75th) ^2^	6.4 ± 5.6, 6, 1–10	6.0 ± 4.8, 5, 2–9	5.7 ± 3.8, 6, 3–8	6.1 ± 3.9, 5, 3.2–8	0.98

^1^One-way ANOVA, ^2^ Kruskal-Wallis test, and ^3^ Fisher’s exact test were used for statistical calculations, and a p-value less than 0.05 was regarded as significant

*BMI* body mass index, *ANC* antenatal care, *SD* standard deviation, *P* percentile

All participants were Thai

### Associations between human milk composition and the duration of lactation

#### Macronutrients

**Fig. 1:**
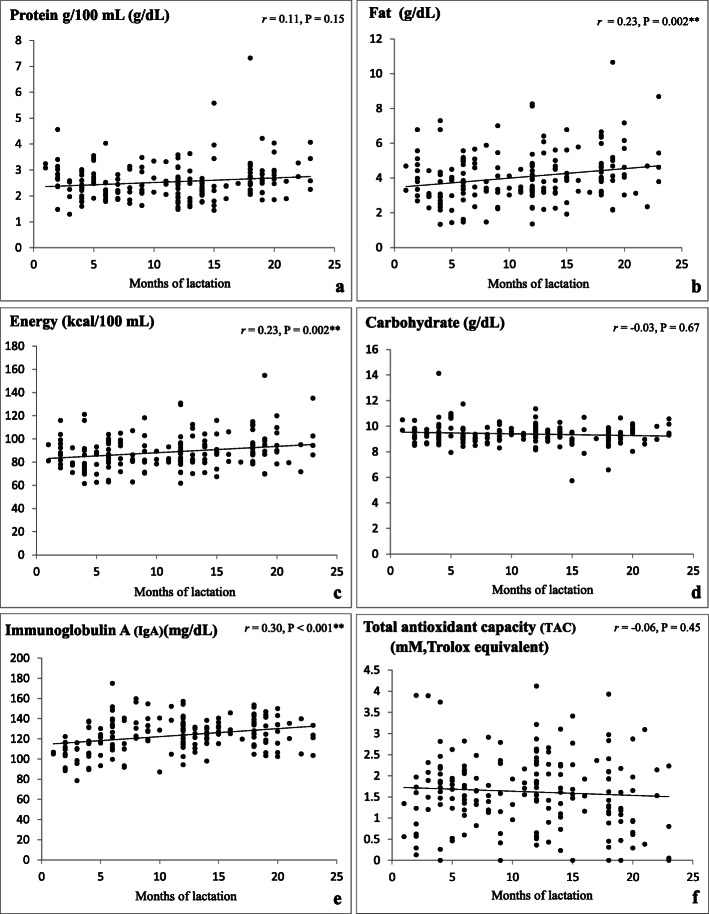
**a**-**f** Correlations between months of lactation and macronutrients, IgA, and TAC of Human Milk. Note: Protein, fat, energy, and carbohydrate content data were analyzed using Spearman’s rank correlation coefficient, while IgA and TAC data were analyzed using Pearson’s correlation coefficient, *p < 0.05, **p < 0.01. All data including outliers in the data set were used in the statistical analysis

The fat and energy contents in HM expressed by mothers who had been lactating for 1–24 months showed a positive correlation with the duration of lactation (*r* = 0.23, *p* = 0.002 and *r* = 0.23, p = 0.002, respectively) (Fig. 1b, c). No significant correlations were found between protein and carbohydrate concentrations and the length of lactation (*r* = 0.11, *p* = 0.15 and *r* = − 0.03, *p* = 0.67, respectively) (Fig. 1a, d). When analyzed as four groups based on breastfeeding duration (Table 2), the protein concentration in HM was higher at 18–24 months postpartum (2.84 ± 0.90 g/dL) than at 6–12 and 12–18 months postpartum (2.39 ± 0.52 g/dL, *p* = 0.001 and 2.40 ± 0.75 g/dL, *p* < 0.001, respectively). The fat and energy contents were significantly higher in HM collected at 18–24 months (4.64 ± 1.61 g/dL and 94.64 ± 16.13 kcal/dL, respectively) than in the other groups (1–6 and 12–18 months, fat concentration 3.67 ± 1.30 g/dL, *p* < 0.001 and 3.90 ± 1.32 g/dL, *p* = 0.03, respectively; energy content 84.86 ± 12.93 kcal/dL, p = 0.001 and 87.91 ± 13.23 kcal/dL, p = 0.03, respectively).

**Table 2:** Comparison of macronutrients, IgA, and TAC in human milk by breastfeeding duration

Breastfeeding Duration	1–6 months (n = 43) ^a^		6–12 months (n = 47) ^b^		12–18 months (n = 50) ^c^		18–24 months (n = 44) ^d^		Mean rank or Mean diff. (SE)	p-value
	Mean ± SD	Median, P25^**th**^–75th	Mean ± SD	Median, P25^**th**^–75th	Mean ± SD	Median, P25^**th**^–75th	Mean ± SD	Median, P25^**th**^–75th		
Protein (g/dL)	2.56 ± 0.62	2.53, 2.21–2.92	2.39 ± 0.52	2.23, 2.05–2.69	2.40 ± 0.75	2.36, 1.87–2.61	2.84 ± 0.90	2.66, 2.29–3.13	50.49, 40.94 ^ab^ 52.22, 42.51 ^ac^ 40.23, 47.68 ^ad^ 50.09, 47.98 ^bc^ 37.34, 55.25 ^bd^ 38.87, 57.31 ^cd^	< 0.001 ^1^ 0.08 ^2^ 0.08 ^2^ 0.17 ^2^ 0.71 ^2^ 0.001 ^2^ < 0.001^2^
Fat (g/dL)	3.67 ± 1.30	3.79, 2.92–4.33	3.96 ± 1.36	3.53, 3.17–4.83	3.90 ± 1.32	3.85, 3.17–4.68	4.64 ± 1.61	4.61, 3.8–5.13	41.26, 49.38 ^ab^ 42.78, 50.63 ^ac^ 34.69, 53.10 ^ad^ 49.20, 48.81 ^bc^ 40.88, 51.47 ^bd^ 41.67, 54.13 ^cd^	< 0.001^1^ 0.14 ^2^ 0.16 ^2^ < 0.001 ^2^ 0.94 ^2^ 0.06 ^2^ 0.03 ^2^
Energy (kcal/dL)	84.86 ± 12.93	86.09, 77.42–91.55	87.77 ± 13.61	83.54, 79.94–96.55	87.91 ± 13.23	86.70, 79.94–95.01	94.64 ± 16.13	94.28, 86.24–99.48	41.26, 49.38 ^ab^ 42.77, 50.64 ^ac^ 34.67, 53.11 ^ad^ 49.20, 48.81 ^bc^ 40.88, 51.47 ^bd^ 41.67, 54.13 ^cd^	< 0.001^1^ 0.14 ^2^ 0.16 ^2^ 0.001 ^2^ 0.94 ^2^ 0.06 ^2^ 0.03 ^2^
Carbohydrate (g/dL)	9.62 ± 1.04	9.43, 8.97–10.12	9.34 ± 0.59	9.27, 8.97–9.71	9.31 ± 0.84	9.35, 8.95–9.74	9.39 ± 0.81	9.41, 8.96–9.73	48.90, 42.39 ^ab^ 49.73, 44.65 ^ac^ 46.53, 41.52 ^ad^ 48.12, 49.83 ^bc^ 44.83, 47.25 ^bd^ 47.48, 47.52 ^cd^	0.65 ^1^ 0.24 ^2^ 0.36 ^2^ 0.35 ^2^ 0.76 ^2^ 0.66 ^2^ 0.99 ^2^
IgA (mg/dL)	110.82 ± 14.06	111.48, 103–117.63	129.59 ± 16.67	130.33, 115.03–140.5	124.29 ± 10.80	125.07, 116.35–131.37	127.16 ± 14.59	126.06, 115.73–139.57	− 18.77 (3.18) ^ab^ − 13.47 (3.14) ^ac^ − 16.34 (3.23) ^ad^ 5.31 (3.06) ^bc^ 2.43 (3.16) ^bd^ − 2.87 (3.12) ^cd^	< 0.001^3^ < 0.001 ^4^ < 0.001 ^4^ < 0.001^4^ 0.08 ^4^ 0.44 ^4^ 0.36 ^4^
TAC	1.61 ± 0.94	1.62, 1.2–2.04	1.61 ± 0.67	1.64, 1.89–2.05	1.84 ± 0.85	1.84, 1.38–2.38	1.60 ± 0.97	1.27, 0.69–2.09	0.03 (0.18) ^ab^ − 0.23 (0.18) ^ac^ 0.21 (0.18) ^ad^ − 0.23 (0.18) ^bc^ 0.18 (0.18) ^bd^ 0.44 (0.18) ^cd^	0.09 ^3^ 0.85 ^4^ 0.19 ^4^ 0.25 ^4^ 0.19 ^4^ 0.33 ^4^ 0.01 ^4^

^1^ Kruskal-Wallis test, ^2^ Mann-Whitney U test, ^3^ one-way ANOVA post hoc test, and ^4^ independent-sample T test were used for statistical calculations

Total antioxidant capacity (TAC) unit is mM, Trolox equivalent; *SD* standard deviation; *P* percentile; *Mean diff.* mean difference; *SE* standard error

^a^HM of the mother who lactation duration between 1-6 months

^b^HM of the mother who lactation duration between 6-12 months

^c^HM of the mother who lactation duration between 12-18 months

^d^HM of the mother who lactation duration between 18-24 months

#### Immunoglobulin a

The concentration of IgA in HM showed a positive correlation with the duration of lactation (r = 0.30, *p* < 0.001) (Fig. 1e). Of the four groups, the mean IgA concentration was lowest at 1–6 months (110.82 ± 14.06 g/dL) compared with the longer-duration groups (6–12, 12–18, and 18–24 months of lactation; 129.59 ± 16.67, 124.29 ± 10.80, and 127.16 ± 14.59 g/dL, respectively, p < 0.001, Table 2).

#### Total antioxidant capacity

The antioxidant capacity of HM showed no significant correlation with the lactation duration lactation (r = − 0.06, *p* = 0.45) (Fig. 1f).

#### Factors affecting human milk composition

Multiple linear regression analysis was used to assess the association between the months of lactation and milk composition with maternal age, maternal BMI, birth order, and breastfeeding frequency as covariates (Table 3). After adjusting for covariates, the number of months of lactation was positively associated with fat concentration (B = 0.31, SE = 0.09, *p =* 0.001), energy content (B = 3.11, SE = 0.92, p *=* 0.001), and IgA (B = 4.17, SE = 1.08, *p <* 0.001) but negatively associated with the carbohydrate concentration (B = − 0.22, SE = 0.01, *p =* 0.04). In addition, maternal BMI was positively associated with the fat concentration (B = 0.09, SE = 0.02, p *<* 0.001) and energy content (B = 0.88, SE = 0.24, p *<* 0.001) but negatively associated with the carbohydrate concentration (B = − 0.04, SE = 0.01, *p =* 0.02).

**Table 3:** Associations between months of lactation, maternal age, maternal BMI, birth order, breastfeeding frequency, and human milk composition using multiple linear regression

	Protein (g/dL)		Fat (g/dL)		Energy (kcal/dL)		Carbohydrate (g/dL)		IgA (mg/dL)		TAC (mM)	
	B (SE)	95% CI	B (SE)	95% CI	B (SE)	95% CI	B (SE)	95% CI	B (SE)	95% CI	B (SE)	95% CI
Months of lactation	0.09 (0.05)	− 0.01, 0.19 (*p* = 0.06)	0.31 (0.09)	0.13, 0.49 (p = 0.001)	3.11 (0.92)	1.30, 4.92 (p = 0.001)	− 0.22 (0.01)	− 0.22, − 0.01 (*p* = 0.04)	4.17 (1.08)	2.03, 6.31 (p < 0.001)	− 0.04 (0.06)	− 0.15, 0.08 (*p* = 0.54)
Maternal age	− 0.01 (0.01)	− 0.03, 0.02 (*p* = 0.62)	− 0.03 (0.02)	− 0.08, 0.01 (*p* = 0.18)	− 0.32 (0.23)	− 0.78, 0.14 (*p* = 0.17)	0.02 (0.01)	− 0.004, 0.05 (*p* = 0.09)	− 0.40 (0.28)	− 0.95, 0.14 (*p* = 0.15)	0.03 (0.02)	− 0.01, − 0.05 (*p* = 0.10)
Maternal BMI	0.02 (0.01)	− 0.003, 0.048 (p = 0.09)	0.09 (0.02)	0.04, 0.14 (*p* < 0.001)	0.88 (0.24)	0.40, 1.36 (p < 0.001)	− 0.04 (0.01)	− 0.06, − 0.01 (*p* = 0.02)	−0.40 (0.29)	− 0.96, 0.14 (p = 0.17)	−0.02 (0.02)	− 0.05, 0.02 (*p* = 0.35)
Birth order	−0.02 (0.09)	− 0.19, 0.16 (*p* = 0.85)	0.19 (0.16)	− 0.13, 0.52 (*p* = 0.25)	1.92 (1.64)	− 1.33, 5.16 (p = 0.25)	− 0.22 (0.16)	−0.22, 0.27 (*p* = 0.77)	− 0.15 (1.94)	−3.98, 3.69 (*p* = 0.94)	−0.003 (0.11)	− 0.21, 0.20 (*p* = 0.98)
Breastfeeding frequency	−0.01 (0.01)	− 0.04, 0.01 (*p* = 0.27)	−0.001 (0.023)	− 0.05, 0.04 (*p* = 0.97)	−0.01 (0.23)	− 0.46, 0.44 (p = 0.97)	−0.04 (0.02)	− 0.04, 0.01 (*p* = 0.39)	−0.20 (0.27)	− 0.73, 0.33 (*p* = 0.45)	0.02 (0.02)	− 0.004, 0.05 (p = 0.10)

A *p*-value less than 0.05 was regarded as significant. *CI* confidence interval, *B* unstandardized beta, *SE* standard error

Maternal age, maternal BMI, birth order, and breastfeeding frequency were analyzed as covariates

## Discussion

We reported that the number of months of lactation was positively associated with fat, energy, and IgA contents but negatively associated with carbohydrate concentration in HM. No association was found between duration of lactation and protein concentration or TAC. Maternal BMI also had an association with HM composition in our study*.*

The demographic or sample characteristics of our participants did not show significant differences between groups based on breastfeeding duration. However, the participants’ education level was higher than in the general Thai population. In Thailand, socioeconomic differences are prominent. As the recruitment process was limited to populations in urban areas and populations with internet access, those who were informed about the project were more likely to have higher education levels than individuals in the general population. Breastfeeding frequency was not significantly different by breastfeeding duration. Our results are consistent with those of previous studies [12, 13] reporting no significant differences in the mean number of breastfeeding episodes per day. Mandel et al. [12] reported feeding frequencies of 7.1 and 5.9 feedings/day for a short-duration group (6–12 months) and a long-duration group (12–39, respectively. Shehadeh et al. [13] reported mean lactation frequencies of 7.1 and 6.9 feedings/day for participants with breastfeeding durations less than one year and longer than one year, respectively. These results can be explained by the need to maintain frequent nursing throughout the lactation phases to preserve the milk supply.

### Macronutrients

We observed that protein concentration was not related to lactation duration. In contrast, two recent studies demonstrated that protein concentration significantly increased during the second year postpartum [14, 15]. In 2018, Czosnykowska-Lukacka et al. [14] reported a positive correlation between lactation duration and the concentration of protein and true protein in milk expressed by 136 mothers who had lactated from 1 to 48 months postpartum (r = 0.44; *p* < 0.05 and r = 0.45; p < 0.05, respectively). In 2016, Perrin et al. [15] described longitudinal changes in HM composition in the second year postpartum. They recruited 19 lactating women who were 9–11 months postpartum at the time of enrollment and provided monthly milk samples until at least 18 months postpartum. This study showed that the total protein concentration increased in the second year postpartum and contained higher protein concentrations than pooled milk samples from 51 donors with breastfeeding duration less than one year.

We observed that fat and energy contents were positively associated with the duration of lactation. This is consistent with previous results reported by Mandel et al. [12] who demonstrated that HM expressed by mothers who had been lactating for more than one year (12–39 months) showed a significant increase in fat content compared with milk expressed by mothers who had been lactating for shorter periods (6–12 months). Czosnykowska-Łukacka et al. [14] showed that the fat content significantly increased in HM expressed by mothers lactating beyond 18 months postpartum, whereas Shehadem et al. [13] and Perrin et al. [15] concluded that fat concentration was not related to the lactation duration.

A negative association between carbohydrate concentration and the duration of lactation was observed in this study. Few prior studies have examined the carbohydrate concentration in HM beyond the first year, and results are inconclusive. Czosnykowska-Łukacka et al. [14] showed that the carbohydrate content was significantly lower in women lactating for 12–18 months compared with women lactating for 1–12 months, while no change was reported by others [13, 15].

We also assessed the macronutrient composition of HM across four periods of breastfeeding duration. We observed that the protein concentration in HM after 18 months postpartum was significantly higher than in HM collected at 6–12 and 12–18 months postpartum. The fat and energy contents were higher in HM after 18 months than in the other groups (1–6 and 12–18 months of lactation). This variation may be due to decreases in volume and mammary gland involution during the weaning process, which regularly occur during longitudinal breastfeeding. Garze et al. [16] reported that protein and fat concentrations increased during weaning. Neville et al. reported a significant increase in protein, but a decrease in the lactose concentration was observed during gradual weaning when the milk volume was below 400 mL/day [17].

### Immunoglobulin a

Significantly higher levels of IgA were observed during extended lactation lasting up to two years postpartum. Similar to our results, Perrin et al. [15] reported that the IgA concentration gradually increased (*p* < 0.05) over a study period of 11–17 months postpartum. Other results remain inconclusive. Prentice et al., 1984 [18] measured the concentration of IgA in the HM of 153 rural Gambian mothers who lactated from 14 days to 26 months postpartum and described that IgA concentrations decreased significantly (*p* < 0.001) during the first year of lactation. On the other hand, Hennart et al., 1991 [19] observed that the concentration of IgA remained stable throughout 18 months of lactation. They also compared IgA concentrations in 54 milk samples from urban mothers with 73 milk samples from rural mothers, with IgA concentrations significantly higher in samples from rural mothers (*p* < 0.05). The study also found that urban mothers had substantially higher milk yields (612 ± 27 mL/day) than rural mothers (307 ± 16 mL/day), and a significantly higher mean breastfeeding frequency among urban mothers (10.1 times/day) than rural mothers (6.8 times/day, p < 0.05).

We observed that the mean IgA concentrations varied between the four groups based on the breastfeeding period, with the lowest concentration observed in the 1–6 month group compared with the longer-duration groups. This variation may have been observed because our samples were collected from different women, and we did not control for factors potentially influencing the IgA level in these grouped analyses, such as breastfeeding frequency, milk output per day, geographical region (rural or urban area), maternal nutritional status, and the stage of lactogenesis (weaning and non-weaning) [17–20].

### Total antioxidant capacity

The results of our study showed that TAC was not related to lactation duration. A few studies have focused on the relationship between TAC in breast milk and postnatal age. In 2009, Zarban et al. [21] measured TAC in 115 healthy mothers of full-term infants at five timepoints: in colostrum at 2 ± 1 days after birth (*n* = 115), transitional milk at 7 ± 3 days (*n* = 97) and 30 ± 3 days (*n* = 102), and mature milk at 90 ± 7 days (*n* = 100) and 180 ± 10 days after birth (*n* = 91). They reported that TAC was significantly higher in colostrum than in transitional and mature milk [21]. The same TAC pattern was reported by Quiles et al. [22] who evaluated changes in TAC in HM during the first month of lactation. Based on limited research, the highest levels of antioxidant components and TAC can be concluded to be present in colostrum, which then decrease during early lactation [21–23]. To the best of our knowledge, the present study is the first to describe TAC in breast milk from women up to two years postpartum, with no observed change in the antioxidant capacity over time.

### Factors affecting the milk composition

We investigated potential predictors of the HM composition, including the months of lactation, maternal age, maternal BMI, birth order, and breastfeeding frequency*.* Apart from the months of lactation as discussed above, the only factor showing an association with HM composition in our study was maternal BMI, which was significantly positively associated with the fat concentration and energy content. A positive correlation between fat content and maternal BMI has been repeatedly reported [24–27], while information on the association between maternal BMI and carbohydrate content remains limited and inconclusive*.* Similar results have been shown by Chang et al. [27]. The authors measured the concentrations of macronutrients in 2632 mature breastmilk samples *(*1–8 months postpartum*)* and found that maternal BMI was negatively associated with lactose content. HM is a dynamic fluid that can vary in composition according to maternal diet. Elucidating the reason for the association between maternal BMI and HM macronutrient content is difficult because diet type and eating behavior may differ for each group *(*normal or abnormal BMI*).* However, most studies have reported that maternal diet has a slight effect on the contents of many nutrients in HM [25, 28, 29]*.*

### Study limitations

Some limitations should be noted. First, the average education level of our participants was higher than that of the general population. Applying the findings of this study to the general population would require further investigation. Second, the underlying health conditions of the mothers and their offspring were evaluated by self-report questions without medical documentation. Third, milk volume [19], genetic variation [8, 30], and environmental factors such as dietary intake, the time since the last feeding, and ethnicity [31, 32] have been shown to influence HM composition, and these factors were beyond the scope of this study. Future research should include a prospective cohort study to reduce individual bias at each time point with careful adjustments for the potential effects of such factors.

## Conclusion

The month of lactation was found to be positively associated with the fat, energy, and IgA levels in HM for up to two years postpartum and negatively associated with carbohydrate concentration. More prospective cohort studies are needed to obtain evidence-based knowledge regarding the changes in HM composition throughout the course of lactation.

## Data Availability

The data sets used and/or analyzed during the current study are available from the corresponding author on reasonable request.

## Abbreviations

BMI: Body mass index
HM: Human milk
IgA: Immunoglobulin A
TAC: Total antioxidant capacity
TEAC: Trolox equivalent antioxidant capacity
